# Evaluation of the Prevalence of Hepatitis B, Hepatitis C, and HIV in Inmates with Drug-Related Convictions in Birjand, Iran in 2008

**Published:** 2010-03-01

**Authors:** Zohreh Azarkar, Gholamreza Sharifzadeh

**Affiliations:** 1 Department of Infectious Disease, Birjand University Medical Sciences, Birjand, Iran; 2 Department of Epidemiology, Birjand University Medical Sciences, Birjand, Iran

**Keywords:** HBV, HCV, HIV, Prisoner, Drug-Related Crimes

## Abstract

**Background and Aims:**

Hepatitis B virus (HBV), hepatitis C virus (HCV), and human immunodeficiency virus (HIV) are common infections among prisoners. Addicted prisoners are at a higher risk than the normal population for contracting these diseases. Many studies have reported higher prevalence rates of HBV, HCV, and HIV in prisoners. Because of this problem, this study was conducted to evaluate the serologic prevalence of these three diseases in prisoners convicted of drug-related crimes.

**Methods:**

A descriptive cross-sectional study was conducted on a random sample of prisoners with drug charges who were inmates in a prison in Birjand, Iran. Information was collected via questionnaire after obtaining prisoners’ informed consent and blood samples were tested for hepatitis B surface antigen (HBsAg), antibodies to HCV (anti-HCV), and antibodies to HIV (anti-HIV). The results were analyzed by chi-square tests.

**Results:**

In this study, 358 prisoners were selected. 80.2% of prisoners were male, and 19.8% were female. The average age was 34.7±12 years. 39.1% were addicted to drugs, 54.2% were smokers, and 19.3% had tattoos. 8.4% had had extramarital intercourse, and 16.8% had had a sexually transmitted disease (STD) in past. HBsAg, anti-HCV, and anti- HIV prevalence in these samples were 6.1%, 8.1%, and 0%, respectively.The prevalence rate of HBV in the addicted prisoners was 4.3%, and the rate in non-addicted prisoners was 7.3% (P = 0.24).The prevalence of HCV in addicted prisoners was 15.7%, and the prevalence in non-addicted prisoners was 3.2%; this difference was significant (P < 0.001).Furthermore, a significant difference between the prevalence of HBV and extramarital intercourse was noted (P < 0.005).A significant difference between HCV and transfusion, history of STDs, addiction, and tattooing was noted.

**Conclusions:**

The survey showed that HCV, HBV, and HIV prevalence rates in prisoners were 8.1%, 6.1%, 0%, respectively. The prevalence rates of HCV and HBV in addicted prisoners were 15.7% and 4.3 %, respectively. Studies performed in Iran and other countries have shown that the prevalence rates of HBV, HCV, and HIV in addicted prisoners were higher than the rates in non-addicted prisoners. These results indicate that HBV, HCV, and HIV are significant problems in prisons, and efforts to reduce the risk of these infections, such as education and vaccination, should be considered.

## Introduction

Addiction to illegal drugs has posed itself as a major global problem and has damaged many families and communities beyond repair. Iran is subject to a flood of opium due to its extensive borders with the foremost of international drug producers: Afghanistan and Pakistan. There are 2 million drug addicts and 800 thousand recreational drug users in Iran, which amounts to 4% of the Iranian population [1].The 1950s marked the appearance of injecting drug users (IDUs), and consequently the femergence of new problems, particularly infectious diseases. Infection is the most prevalent cause of death among drug addicts. Most IDUs share syringes and needles for injection. Most of them are friends or relatives or simply strangers given shared syringes in injection galleries. These galleries are secret places where instruments of injection are lent to IDUs. The IDUs obtain their drugs from these galleries as well. These places are especially risky for the transmission of infectious agents [2].Nowadays, addiction, particularly injection, is the greatest risk factor for blood-borne viral diseases such as Hepatitis B, Hepatitis C, and acquired immune-deficiency syndrome (AIDS) [3].In a study conducted on 479 addict prisoners in Hamadan, Iran in 2002, 58.4% were non-IV drug abusers, and the frequencies of antibody to hepatitis C virus (HCV Ab), hepatitis B surface antigen (HBsAg), and antibody to human immune deficiency virus (HIV Ab) were 7.35%,1.46%, and 1.04%, respectively [4].Epidemiologically, addicts (IDUs in particular) constitute the second-largest population of AIDS patients after homosexuals. The two populations who are most susceptible to HIV infection are prison inmates and individuals who share  injection instruments [5].Hepatitides are another complication of injecting addictions. Parallel alcohol consumption in hepatitis patients has been found to accelerate the process of hepatic damage and its progression to cirrhosis. Viral hepatitides B, C, and D are transmissible through shared syringe needles [2][6].There are 200 million cases of hepatitis C worldwide, and IDUs constitute 42% of patients suffering from the disease, most of whom progress to chronic hepatitis. Injecting addictions play a greater role in transmission of HCV than does sexual activity [2]. Indeed, it is believed that the most important and prevalent route of transmission for this disease is intravenous injection among drug abusers. Seventy-eight percent of IDUs develop hepatitis C after 1 year, 83% after 5 years, and 94% after 10 years or more [7][8].In another Iranian study, this one conducted on 312 addicted male prisoners 2002, the prevalence of HCV infection was found to be 30.8%. The major risk factors in this sample were duration of addiction, duration of imprisonment, length of alcohol consumption , having multiple sexual partners, route of drug use, and type of drug [9].There are 400 million cases of hepatitis B infection worldwide. Two million people die each year from complications of hepatitis B, most notably hepatic cirrhosis and hepatocellular carcinoma [10].In a study conducted in Nahavand, Iran in 2004, a history of surgery and a history of incarceration were the most common risk factors for HBV infection [11].Sixty percent of Iranian prisoners have been condemned for drug-related crimes, and 15-20% of other crimes are also associated with addiction and drug trafficking.In short, prison inmates are at high risk of contracting hepatitis B, hepatitis C, and HIV due to high frequency of drug abuse and unsafe sexual activities.  Different studies have suggested serologic evidence of HCV infection in one out of three prisoners [12][13][14]. Despite these realities, inmates are not routinely screened for these infections. There is an annual screening for AIDS among prisoners in Iran, but little is known about hepatitis infections, especially hepatitis C [15].This study was conducted to evaluate the serologic prevalence of hepatitis B, hepatitis C, and HIV in prisoners convicted of drug-related crimes. These infections were compared among addict and non-addict inmates.

## Materials And Methods

This analytical-descriptive study was conducted on 358 prisoners with drug-related convictions in Birjand, Iran. The cases were selected randomly from the list of prisoners' codes. Subsequently, anonymous questionnaires based on the confidential codes of the prison authorities, including prisoners' demographic data and risk factors, were completed by the public health experts through interviews with prisoners. Then, 5 mL of blood were drawn from each prisoner to be screened for hepatitis B, hepatitis C, and HIV. The samples were evaluated at the Blood Transfusion Center by enzyme-linked immunosorbent assay (ELISA). The collected data were entered into SPSS software and analyzed with chi-square tests, fisher's exact tests, and regression logistics at α = 0.05.

## Result

This study included 358 prisoners with drug-related sentences. The average age was 34.7 ± 12 years. Two hundred eighty seven (80.2%) of cases were men and 71 (19.8%) were women. One hundred forty (39.1%) were addicted to drugs, and the most frequent routes of intake were inhalation (62.9%) and multiple routes (8.5%) (Table 1). The most frequently used substance was opium (102 cases; 72.9%). Among all subjects, 19.3% (69 cases) had a history of tattoos, 54.2% (194 cases) were cigarette smokers, 8.4% (30 cases) had had extramarital intercourse, 30.4% (109 cases) had a history of surgery, 8.4% (30 cases) had had a blood transfusion, and 16.8% (60 cases) had a history of sexually transmitted diseases (STDs).The prevalence rate of hepatitis B was 6.1% (22 cases), the rate of hepatitis C was 8.1% (29 cases), and the rate of HIV was zero. Statistically, there was a significant difference between the prevalence of hepatitis C among addict and non-addict prisoners (Table 2).Moreover, it was indicated that the prevalence of hepatitis C was significantly associated with a history of surgery, blood transfusion, STDs, tattooing, and extramarital intercourse (P < 0.05) (Table 3).The prevalence of hepatitis B was significantly associated with extramarital intercourse; specifically, 26.7% (14 cases) of men with a history of extramarital intercourse and 4.3% (8 cases) of men without a history of extramarital intercourse (P < 0.001) had HBV (odds ratio [OR] = 8.16; 95% confidence interval [CI], 3.1-21.5)The prevalence of hepatitis B was 7% (20 cases) in men and 2.8% (2 cases) in women (P = 0.27), and the prevalence of hepatitis C was 8.7% (25 cases) in men and 5.6 % (4 cases) in women (P = 0.39); these differences were not significant. Similarly, the prevalence of hepatitis B was not significantly associated with surgery, blood transfusion, or STDs.

**Table 1:** Frequency of type of addiction in addict prisoners.

**Type of Addiction**	**Number**	**Percentage**
Injection	5	3.6
Inhalation	88	62.9
Oral	13	9.3
Both Inhalation and Oral	22	15.7
Inhalation ,Oral, and Injection Together	12	8.5
Total	140	100

**Table 2:** Comparison of HBV and HCV prevalence in addict and nonaddict prisoners.

**Type of Addiction**	**Addict N = 140**		**Non-addict N = 218**		**P-value Chi-square Test**	**Risk Estimate OR (95% CI)**
**Hepatitis**	**Number**	**Percent**	**Number**	**Percent**		
HBV+	6	4.3	16	7.3	P =0.24	0.6(0.22-1.48)
HCV+	22	15.7	7	3.2	P<0.001^a^	5.6(2.3-13.5)

^a^ α = 0.05

**Table 3:** Frequency of HCV in prisoners according risk factors.

	**HCV**	**Positive**		**Negative**		**P-value Chi-square or Fisher’s Exact Test**	**Risk Estimate OR(95%CI)**
**Risk factor**		**Number**	**Percent**	**Number**	**Percent**		
Surgery n = 109		18	16.5	91	83.5	P<0.001^a^^c^	4.3(1.9-9.4)
Transfusion n = 30		8	26.7	22	73.3	P=0.001^a^^b^	5.3(2.1-13.4)
STD n = 60		9	15	51	8.5	P = 0.04 b	2.4(1.1-5.7)
Tattooing n = 69		13	18.8	56	81.2	P<0.001^a^^c^	3.96(1.8-8.7)
Extramarital Intercourses n = 30		15	50	15	50	P<0.001^a^^b^	22.4(9.2-54.8)

^a^ α = 0.05

^b^ Fisher’s Exact Test

^c^ Chi-square

## Discussion

Drug addiction brings about serious and severe problems and jeopardizes social and individual health. The most horrendous facet of this problem is injection. The problems and diseases that occur as a result of injection can easily lead to a person's death.The findings of this study indicated that 39.1% of prisoners convicted of drug-related crimes were drug abusers themselves. Moreover, 6.1% of inmates were infected with hepatitis B, and 8.1% were infected with hepatitis C. The prevalence of HIV was zero in our study.The prevalence of hepatitis C was considerably higher in addicts than in non-addicts (15.7% compared to 3.2%). Because these infections are transmissible through infected needles and syringes shared by IDUs, it is expected that IDUs should have a higher prevalence of these infections than the general population, particularly in the case of hepatitis C. For instance, in a study conducted on IDU prisoners in Mashad, Iran in 2002, 60% were infected with hepatitis C, 3% with hepatitis B, and 7% with HIV [16]. In a study conducted on addict prisoners in Khazarabad, Iran in 2001, tattooed inmates were shown to have at least 3.5 times higher risk of infection with hepatitis C. Moreover, 30.8% of the cases were found to have hepatitis C, 81.7% of which were IDUs [15].Indeed, tattooing may be a major route of HCV transmission. Our study indicates that the prevalence of hepatitis C is significantly related to STDs, tattooing, and extramarital intercourse.In another study conducted on prisoners of Sistan and Baluchestan, Iran in 2001, 8.4% of prisoners were HBsAg positive and 9.1% had hepatitis C infection [17]. In a study conducted on high-risk groups in Bushehr, Iran in 2000, the rates of HBV and HCV infection among IDU prisoners were reported to be 16.7% and 52.4%, respectively [18].A study conducted in Iran, among 5,317 prisoners from 2001 to 2005 indicated a significant association between HBV infection and drug abuse in two out of every seven cases [4].Another study conducted on 479 addict prisoners in Hamadan, Iran, the frequencies of HCV Ab, HBsAg and HIV Ab were 7.35%, 1.46%, and 1.04%, respectively. The relationship between being an IDU and antibody to HCV (anti-HCV) positivity was statistically significant [4].Studies conducted in Iran have reported a greater prevalence of hepatitis B, hepatitis C, and HIV infection compared to our study, most likely because those studies were limited to IDU prisoners. Other studies around the world have indicated a higher prevalence of all three diseases in drug addicts than in the general population.In a study conducted on IDUs in Ireland, the prevalence rates of HIV, HBV, and HCV were reported to be 24.5%, 9%, and 3.4%, respectively [3], which is expected because that study used a larger sample and was limited to drug addicts.In a study conducted in the Texas-Mexico border on 320 anti-HCV positive and 307 anti-HCV negative cases, definite and potential risk factors for hepatitis C transmission were evaluated. The multivariable analysis indicated tattooing, drug abuse, and blood transfusion as independent and significant risk factors for hepatitis C transmission [19].In a study conducted on 433 inmates in a Bologna prison, Italy, infection rates of HIV, HBV, and HCV were reported to be 12.5%, 8.1%, and 31.3%, respectively. Drug addiction was common among the inmates, and 33.9% of them were IDUs [20].A 5-year study in a tuberculosis clinic in Moscow reported addiction (76%), hepatitis B, and C (77%) as key pathological agents in association with tuberculosis and AIDS [21].In our study, hepatitis C was significantly more prevalent in drug addicts than in non-addicts.In a nationally representative survey of 1,193 Irish prisoners, 21% had experienced their fist injection in prison, and 71% shared needles. The results of the study suggested that 40% of prisoners in the republic of Ireland used drugs [22].Other studies in Brazil and Glasgow indicated that hepatitis C infection was related to the duration of addiction and imprisonment [23][24].When a drug addict feels a desire for drugs, he or she will be willing to do almost anything to fulfill his or her desire. In prisons, where few people have access to instruments of drug consumption, it is quite likely that inmates will tend to perform risky actions such as sharing infected syringes and needles. Many people who did not inject previously might be tempted to take up the habit of injection due to the limited options in prisons and the method's rapid effects. These facts underline the dangerous role of prisons in infecting prisoners with HIV and hepatitis.Not only is an addict prisoner susceptible to perilous diseases such as AIDS and hepatitis, but he also may function as a vehicle for transmission of causative agents.A study evaluating the effects of interventions such as providing abundant needles for IDUs from 1990 to 2001 reported a decrease in the prevalence rates of HIV and HCV from 54% to 13% and from 80% to 63%, respectively [25], highlighting the importance that such interventions could have for our country.

## Conclusions

The findings of our study indicate a high prevalence of HBV and HCV infections in drug addicted prisoners. Unhealthy and risky behaviors such as tattooing, extramarital sexual intercourse, and drug addiction were the most important factors related to infection. Infected prisoners eventually return to their families and communities and can easily spread their infections through risky behaviors. Therefore, routine screening of addicts, especially in prisons, and training inmates about personal health and the necessity of using clean needles, becoming immunized against hepatitis B, and treating their addictions are suggested as pre-emptive measures.Furthermore, informing individuals, particularly the younger generations, of the dangers associated with drug use, even moderate use, is of utmost importance.
